# Mass Immunization and Vaccine Hesitancy in Children and Their Families: A Long and Winding Road Ahead to Address without a Second Thought

**DOI:** 10.3390/vaccines9070752

**Published:** 2021-07-06

**Authors:** Davide Gori, Marco Montalti, Federica Guaraldi

**Affiliations:** 1 Unit of Hygiene, Department of Biomedical and Neuromotor Sciences, Public Health and Medical Statistics, University of Bologna, 40126 Bologna, Italy; marco.montalti7@studio.unibo.it; 2 Pituitary Unit, IRCCS Istituto delle Scienze Neurologiche di Bologna, 40126 Bologna, Italy; federica.guaraldi@yahoo.it

In 2019, vaccine hesitancy (VH), defined by the SAGE working group as “delay in acceptance or refusal of vaccination despite availability of vaccination services” [1] appeared on the WHO list of the top 10 public health threats requiring urgent attention, along with other global issues much more covered by media, such as Ebola virus infection and climate change [2]. This has led several countries worldwide to enact, strengthen or contemplate mandatory and/or recommended infant and childhood immunization to cover this gap.

In January 2020, a new infectious threat, which turned out to become a pandemic, SARS-CoV-2, struck the world [3]. Non-pharmaceutical interventions (NPIs), i.e., face masks, social distancing and lock-down, have represented the only available strategies to contain the virus widespread up to fall of 2020, when ModeRNA and Pfizer announced to the world [4,5] the completion of phase 3 trials and the forthcoming availability of the first anti-COVID vaccines.

This immediately brought back the attention of the scientific community on people’s acceptance of the new vaccine [6,7].

The success of mass vaccination campaigns registered in the first months in the vast majority of high-income countries suggests that the perception of the high risk related to SARS-CoV-2 infection, combined with the strong desire to return to a normal life after long-term restrictions in social life, has overall overcome the understandable reluctance toward the new vaccines, approved after reduced timing of trial phases. At the same time, important differences in VH rates have been identified according to patient geographical origin, age, gender and profession [6].

Today, while phase 2 of COVID-19 mass vaccination campaign in adults is rapidly proceeding, some issues are still debated, including the opportunity of infant and young children vaccination. Some trials performed in adolescents aged 12–15 years old [8,9] have just ended, thus offering the possibility to expand vaccination to this target population.

This special issue called: “Vaccine Hesitancy and Childhood Immunization: Rationales, Issues and Knowledge Gaps” was conceived in this particular context, with the aim of focusing on knowledge and strategies to improve parents’/guardians’ awareness, thus reducing VH and improving vaccination coverage rates in infants and children.

The first issue to be considered is ethical, as discussed by Meta Rus and Urh Groselj [10]. Indeed, it is important to find a so-called “ethical equilibrium” between parental autonomy and the levels of herd immunity achieved in a specific social and geographic context, since, in case of insufficient coverage rates, an institutional intervention with mandatory vaccination policies could be required.

Compulsory vaccination has proved to play a primary role to counteract the progressive decrease in vaccination coverage rates registered in the last decades [11,12,13]. However, as observed by D’Errico et al., the current global policy trend in developed countries is to progressively abandon this strategy, strongly opposed by the so-called “no-vax” groups, who greatly benefit from the ease of content dissemination on new social media [14]. Piedrahita-Valdés et al., after examining through a dedicated algorithm about 1.5 million tweets written in the last decade, reported that we are witnessing an increasing polarization of the content conveyed by the new social media (specifically Twitter) against vaccination [15].

Because of the growing disbelief and skepticism spreading through parents/guardians regarding infant and children vaccination, new strategies aimed at disseminating targeted correct information, together with more efficient programs to report potential adverse events following immunization and, not less important, the formulation of effective “informed refusal” (as suggested by the Latvian experience) are strongly required [13,14,16].

In this regard, it was observed by Fanxing Du et al. that parents who inform themselves using professional sources are significantly more prone to vaccination, while those mostly relying on social media as primary source of information are more likely to be hesitant. These findings also underline how, in order to address VH, an increased access to professional sources related to vaccination is needed, including social media themselves, to retrieve the largest number of detailed information [17].

On the other hand, as analyzed by Louis Torracinta et al., there are involuntary factors limiting access to vaccinations, with special reference to determinants in minorities, that, although often ignored by the public debate, significantly impact vaccine coverage [18].

The importance of vaccination strategies and coverage also for other disorders have become more important in light of the current SARS-CoV-2 pandemic. A potential relationship between Bacillus Calmette Guerìn vaccination and lower incidence of COVID-19 disease was found by Garzon-Chavez in an Ecuadorian sample, hypothesizing a potential protective effect of vaccination campaigns for tuberculosis in SARS-CoV-2 spreading [19], even if these preliminary data need to be confirmed by other studies.

Speaking of VH and confidence towards anti-COVID-19 vaccines, Sallam et al. provided a clear picture of how acceptance rates were widely distributed around the world, ranging from 91.3% in China to 23.6% in Kuwait, confirming the extremely heterogeneous context- and contest-specific nature of this phenomenon [20]. Anti-COVID-19 vaccination attitude has been also investigated in smaller populations of interest. For example, Pastorino et al. reported a 94.7% confidence rate among university students, and underlined the vaccine-specific nature of the phenomenon comparing this rate to the 77.5% of confidence registered for influenza vaccination in the same study population [21]. The complexity of VH description is even more evident from the work of Qunaibi et al. investigating the confidence rates of healthcare workers in general and, more specifically, among Arab healthcare professionals, reporting VH rates ranging from 25.8 to 32.8% [22]. This observation reminds us the extreme complexity and, at the same time, the importance of a detailed evaluation of VH.

Finally, attitude toward vaccination can change depending on whether the procedure is performed on the subject him/herself or on his/her children, since several other concerns could contribute to parental decision. For this purpose, we performed a survey over a large number of parents/guardians of children living in Northern Italy, finding an overall rate of attitude toward vaccination of 60.4%, with higher VH rates in young mothers with low educational level, relying on information found on the web/social media, and disliking mandatory vaccination policies [23]. In addition to studying how socio-demographic determinants could influence the hesitancy increase, Graffigna et al. showed, with a more psychological perspective, how health engagement could also prove to be a relevant factor in terms of impact on the decision to vaccinate or not [24].

The investigation of a multitude of individual and population cultural factors and concerns that contribute to VH is extremely complex, especially considering the ever-evolving nature of the phenomenon. At the same time, to achieve the desirable result, i.e., the resolution/significant reduction of social and health burden associated with transmissible disease through population immunization, all efforts should be devoted to the identification of those aspects that could contribute to successful mass vaccination programs.

We hope that this special issue has contributed to focus on some important issues of the scientific debate on a topic as relevant and delicate as this, starting from the ethical point of view to address facing the new-media issue, filling gaps of knowledge of utmost importance in this historical period.
